# Cost-effectiveness of peer-supported self-management for people discharged from a mental health crisis team: methodological challenges and recommendations

**DOI:** 10.3389/fpsyt.2023.1031159

**Published:** 2023-06-02

**Authors:** Marie Le Novere, Sonia Johnson, Brynmor Lloyd-Evans, Louise Marston, Gareth Ambler, Caroline S. Clarke, David Osborn, Danielle Lamb, Rachael Maree Hunter

**Affiliations:** ^1^Research Department of Primary Care and Population Health, Faculty of Population Health Sciences, University College London, London, United Kingdom; ^2^Divison of Psychiatry, Faculty of Brain Sciences, University College London, London, United Kingdom; ^3^Department of Statistical Science, Faculty of Mathematical & Physical Sciences, University College London, London, United Kingdom; ^4^Department of Applied Health Research, Faculty of Population Health Sciences, University College London, London, United Kingdom

**Keywords:** crisis resolution teams, peer-support, cost-effectiveness, quality adjusted life years, economic evaluation, EQ-5D, mental health

## Abstract

**Background:**

Mental health acute crisis episodes are associated with high inpatient costs. Self-management interventions may reduce readmission by enabling individuals to manage their condition. Delivery of such interventions by Peer Support Workers (PSWs) may be cost-effective. CORE, a randomized control trial of a PSW self-management intervention compared to usual care, found a significant reduction in admissions to acute mental healthcare for participants receiving the intervention. This paper aims to evaluate the cost-effectiveness of the intervention over 12 months from a mental health service perspective. Analysis methods of increasing complexity were used to account for data missingness and distribution.

**Methods:**

Participants were recruited from six crisis resolution teams in England from 12 March 2014 to 3 July 2015 (trial registration ISRCTN: 01027104). Resource use was collected from patient records at baseline and 12 months. The EQ-5D-3L was collected at baseline and 4 and 18 months, and linear interpolation was used to calculate 12-month values for quality-adjusted life-years (QALYs). The primary analysis of adjusted mean incremental costs and QALYs for complete cases are calculated separately using OLS regression. Secondly, a complete-case non-parametric two-stage bootstrap (TSB) was performed. The impacts of missing data and skewed cost data were explored using multiple imputation using chained equations and general linear models, respectively.

**Results:**

Four hundred and forty-one participants were recruited to CORE; 221 randomized to the PSW intervention and 220 to usual care plus workbook. The probability that the PSW intervention was cost-effective compared with the workbook plus usual care control at 12 months varied with the method used, and ranged from 57% to 96% at a cost-effectiveness threshold of £20,000 per QALY gained.

**Discussion:**

There was a minimum 57% chance that the intervention was cost-effective compared to the control using 12-month costs and QALYs. The probability varied by 40% when methods were employed to account for the relationship between costs and QALYs, but which restricted the sample to those who provided both complete cost and utility data. Caution should therefore be applied when selecting methods for the evaluation of healthcare interventions that aim to increase precision but may introduce bias if missing data are heavily unbalanced between costs and outcomes.

## 1. Introduction

Between 1998 and 2012, the number of psychiatric beds in England fell by 39%, shifting activity away from acute services and toward care focused on recovery and self-management for those going through an acute crisis episode (1, 2). Crisis Resolution Teams (CRTs) were introduced in England with the aim of encouraging early discharge from hospital or providing intensive home treatment when possible (3. Evidence suggested that these have been successful in reducing hospital admissions and in turn reducing health service costs (4–6). However, more recent evidence has found that CRTs’ service delivery and organization varies and model fidelity is not high, both in the UK (7) and internationally (8). Naturalistic studies suggest they may not consistently have an impact on hospital admissions to an acute mental health ward (9, 10). This may be related to high relapse rates given around 50% of patients are readmitted to acute care within 1 year of contact with a CRT (11). Self-management interventions, which aim to educate and empower individuals to control or reduce the impact of their condition (12), may be useful in reducing readmission to acute care by enabling individuals to keep the severity of their condition in check following discharge from a CRT. There is evidence to suggest that the delivery of such interventions by Peer Support Workers (PSWs) may be cost-effective (13). PSWs are individuals who have shared experiences with the patients, facilitating their ability to provide support and mentorship to those receiving the intervention (14–16). Studies have found that the benefits of employing PSWs, such as reduction in hospital admission to an acute mental health ward and improvement in other aspects of patients’ lives such as social functioning (17–19), outweighed the costs of employing PSWs (13, 18). PSWs are increasingly commonly employed within the English National Health Service (NHS) mental health services and internationally and are advocated in the mental health implementation guidance for the NHS Long term Plan (20–22). The findings from the CORE trial (23) provide evidence to support this approach, demonstrating significant reduction in admissions to acute mental healthcare for participants receiving the CORE peer-supported self-management intervention compared with the control. To our knowledge, the cost-effectiveness of such an intervention following a mental health crisis has not previously been tested. We therefore carried out an economic evaluation alongside the clinical trial with the aim to calculate the probability that the CORE peer-provided self-management intervention was cost-effective compared to control. The control was Treatment as Usual (TAU) accompanied by a self-management workbook without guidance on how to use it.

Recruitment, retention and follow-up are known issues in clinical trials; loss to follow up may occur if the participant’s state of health, particularly mental health worsens, and they are no longer able to engage with the trial. These issues can be more pronounced in mental health trials, especially those involving complex interventions, where the participant commonly knows if they have been randomized to the intervention or control. Participants randomized to control may lose interest once they know they will not immediately receive the trial intervention (24).

Economic evaluations alongside clinical trials often face a high level of missing cost data due to their reliance on self-reported measures such as the Client Service Receipt Inventory (CSRI) to collect resource use information (25), that ask participants or carers to recall what appointments and other treatments they have had. Trial participants who are missing this type of outcome data may be systematically different from participants with complete data, so to simply ignore the missing data potentially introduces bias. As a result, different methods have been explored in order to minimize missing cost data in economic evaluations (26), including using electronic healthcare records to supplement or replace self-completed questionnaires, and in this study resource use information is collected using medical records from mental health Trusts. While the use of electronic healthcare records has some shortfalls in terms of scope, it reduces the risk of missing data caused by illness, disengagement with the trial, patient recall and questionnaire design (27). Instead, there may now be more missing data on the self-reported health-related quality of life side of the equation, which may affect the interpretation of the results in a different way to missing data on the cost side.

The aim of this paper is to report the 12- month cost-effectiveness of CORE, a peer-provided self-management intervention, compared with the control, where data were collected over 12 months for resource use and 18 months for health-related quality of life. The analysis used data from patient medical records for healthcare resource use in addition to self-completed questionnaires for health-related quality of life to calculate utilities and quality adjusted life years (QALYs). Medical records are considered to be relatively complete, whereas self-completed questionnaires are subject to a larger quantity of missing data. This imbalance in data completeness between costs and outcomes leads to methodological challenges which must be addressed in order to achieve our aim. As a result, in this paper we explore the differential impact of economic evaluation methods of increasing complexity to account for missing data. We also explore the impact of accounting for resource use skew, which, although always present in economic evaluations, is particularly marked in acute crisis care due to the high use of expensive inpatient care.

## 2. Methods

### 2.1. Sample

Participants were identified from caseloads from CRTs in six NHS Trusts in London, South East and South West England from 12 March 2014 to 3 July 2015. Participants were recruited after they were discharged from the CRT and were eligible if they had been on the caseload for at least a week because of a crisis. More detail on the eligibility and exclusion criteria is available elsewhere (23). The study included an internal pilot in which 40 participants were recruited (23).

### 2.2. Treatment offered

Participants and care providers were not blinded but neither were they informed of the participants’ allocation until after they had been discharged from the CRT, to minimize any impact on discharge planning from trial participation. Those in the treatment group were given a personal recovery workbook and offered up to 10 sessions with a PSW, aimed to be completed within 4 months, to support them in the completion of the workbook in addition to usual care. For a more detailed description of the intervention components please see Johnson et al. (23). Those in the control group received usual care and the workbook by post only, without additional guidance.

### 2.3. Measures

#### 2.3.1. EQ-5D-3L

The EQ-5D-3L (28) was collected at baseline and 4 months initially. During the trial, additional funding was received to add a follow-up point for the self-completed questionnaire at 18 months, so EQ-5D-3L was also collected at this point. The formula developed by Dolan (29) and the area under the curve method were used to calculate QALYs for each group from baseline to 4 and 18 months (30). For participants who died during the trial their utility was assessed as 0 at the date of death and a straight line was assumed from their last completed EQ-5D-3L to the time of death. To calculate the mean difference in QALYs and 95% confidence intervals between the intervention group and control, a regression with 5,000 bootstrapped replications was used controlling for group, baseline EQ-5D-3L utility score and clustering by peer support worker (30). For the 18-month analysis, a discount rate of 3.5% was used to discount QALYs from 12 to 18 months in line with NICE guidance (31).

To match the QALY follow-up duration with resource use data collected from clinical records, QALYs were calculated over 12 months using linear interpolation, a straight line between the 4- and 18-month follow-up points, with the value on that line at 12 months assumed to be the utility value that would have occurred at 12 months.

### 2.4. Service utilization and costs

#### 2.4.1. Cost of mental health service use

Acute and community mental health service use for both groups was collected at baseline and 12 months for the previous 12 months from electronic patient records held by mental health Trusts. Unit costs were gathered from published sources including the Personal Social Services Research Unit (PSSRU) (32) and NHS reference costs (33) to be applied to mental health service use over 12 months. The cost of mental health clusters was estimated based on diagnosis. Mental health clustering is used in the UK to allow patients to be grouped together by severity while still allowing a degree of variation in the combination and severity of needs.

#### 2.4.2. Cost of intervention

The cost of training PSWs and supervision by clinical staff was included in the intervention cost. The hourly cost of an ‘Agenda for change’ Band 3 staff member (pay bands used by the NHS, example role: emergency care assistant, occupational therapy support worker) from the PSSRU (32) was used for the hourly cost of a PSW. Costing for supervision was varied by grade and frequency (see Supplementary material), with clinical supervision predominately being provided by Band 8a. The cost of the intervention also included PSWs time providing support based on the number of appointments participants had and the duration of appointments. The cost of the workbook is not included in the intervention costs as both groups received it.

A linear regression with 5,000 bootstrap replications, controlling for baseline service use, and clustered by peer support worker, was used to calculate the mean difference in costs between the intervention and control group and 95% confidence intervals.

As costs were reported for baseline and 12 months only, there was no discounting of costs. All costs reported are in 2015/2016 British Pounds.

### 2.5. Data analysis

The planned primary analysis was a complete-case analysis calculating the incremental cost per QALY gained by dividing the mean difference in costs between the two groups by the mean difference in QALYs found using the linear interpolation for 12-month utility. To account for any potential relationship between costs and QALYs, cost-effectiveness analyses commonly use seemingly unrelated regression (SUR; Stata command SUREG), which account for the relationship through correlated error terms, to calculate mean incremental costs and QALYs (34). This method does not allow for clustering by PSW. Ignoring clustering in randomized trials can lead to biased and incorrect conclusions (35, 36). In the case where a non-pharmaceutical intervention is delivered by multiple health professionals, those participants who are treated by the same health professional may have similarities or be clustered due to differences in the healthcare professionals. This violates the assumption of independence and appropriate statistical methods are needed to account for this (37, 38). As a result, for our original primary analysis, we calculated the mean incremental costs and QALYs using complete-case linear regression controlling for baseline service use and including clustering for PSW with 5,000 bootstrap replications. Regression analyses for costs and QALYs were run separately.

Other methods for use in cost-effectiveness analyses (CEA) of cluster randomized trials include non-parametric two-stage bootstrap (TSB) (35) which accounts for the relationship between the costs and outcomes by sampling the costs and effects in pairs which maintains the relationship between the two in the bootstrapped results (34). The different methods and their benefits and pitfalls were explored in the context of this analysis considering the high levels of missing data present for QALYs. They are laid out in this paper as follows:

- The original separate primary regression analyses
- Sensitivity analyses including joint analysis of costs and QALYs using TSB
- Missing data analyses
- Sensitivity analysis around resource use skew

#### 2.5.1. Original separate primary analyses

##### 2.5.1.1. Incremental cost-effectiveness ratio

The planned primary analysis was a complete-case analysis calculating the incremental cost per QALY gained by dividing the mean difference in costs between the two groups by the mean difference in QALYs found using the assumed 12-month utility. 12 month was chosen as the more conservative option given we have costs at this timepoint and utility before and after. This was considered more robust than extrapolating costs to 18 months (cost data only being available up to 12 months). The analysis was also designed to be aligned with the main statistical analysis which was comparing readmission within 1 year between the two groups using a logistic regression (23).

##### 2.5.1.2. Cost-effectiveness plane and cost-effectiveness acceptability curve

A CEP is used to report the bootstrapped 12-month QALYs and 12-month costs. These results are also reported on a CEAC to show the probability that the intervention was cost-effective compared with the control for a range of cost-effectiveness threshold values from £0 to £100,000, with probabilities reported for a £20,000 cost-effectiveness threshold. We also report the probability that the intervention was cost-effective compared with control for this range for:

- 12-month costs and 18 months QALYs
- 12-month costs and 4 months QALYs

The primary 12-month costs and QALYs analysis was repeated using the non-parametric TSB.

#### 2.5.2. Sensitivity analysis

Uncertainty around the following aspects of the analysis were explored in sensitivity analysis using the TSB method:

i. The cost of the intervention (Supplementary material)

The analysis was repeated using supervision and training costs provided by mental health Trusts to calculate the cost of PSWs as well as exploring how the results might change if supervision was weekly rather than fortnightly.

ii. Calculating 12-month utility

In the primary analyses, we assume that trial participants’ utility changes in a linear way between timepoints. To test the impact of this assumption, the last values were carried forward using utility at 4 months to impute utility at 12 months and recalculating QALYs at 12 months. We then did the same again but with next value carried backward, i.e., using utility at 18 months to impute utility at 12 months. We present these results on a CEAC alongside the estimated 12-month QALY results.

#### 2.5.3. Missing data analysis

Only 52% of participants have complete data for all time points of the EQ-5D-3L. Given high proportions of missing data can lead to misleading results if not dealt with appropriately, we have followed the process laid out by Faria et al. (39), on how to deal with missing data in within-trial CEAs. The process is broken down into 3 stages: descriptive statistics to inform assumptions on the missing data mechanism, choosing an appropriate method to deal with the missing data for the base-case analysis using these assumptions, and finally, sensitivity analysis to explore how the results change with the asssumptions made. The first stage is to explore the data in order to inform whether the data are likely to be missing completely at random (MCAR), missing at random (MAR) or missing not at random (MNAR). The classifications of missing data are explained further in Faria et al. (39).

For the data to be MCAR, missing data must be independent of both observed and unobserved characteristics, although covariate dependent missingness (CD-MCAR) occurs when the probability of missingness is dependent on baseline covariates but is independent of the missing and observed outcome. Data can be MAR if missingness can be accounted for using the observed data and the probability of missingness is independent of unobserved characteristics. MNAR occurs when missingness is dependent on unobserved factors, and this may introduce bias if for example, individuals are more likely to have missing data depending on if they have good or bad outcomes.

To determine the type of missing data present, we used logistic regressions to investigate the relationship between observed variables and missingness. Predictors of missingness in 4- and 18-month EQ-5D-3L data included whether participants were in employment and their level of educational attainment. Being in employment and higher levels of educational attainment were associated with lower levels of missing data. This analysis included the main trial only as the wording of questions changed between the pilot and main trial.

We used logistic regression to test if there was a relationship between missingness and previously observed outcomes and found no association between utility score at 4 months and missing utility data at 18 months. This suggests that there was no association with having a worse or better observed outcome at 4 months and likelihood of missing outcome data at 18 months.

When using linear interpolation to calculate 12-month QALYs, there was 48% missing data for QALYs. Multiple imputation using chained equations (MICE) and predictive mean matching was therefore used to impute 4- and 18-month utility data for 48 imputations, stratified by group. The imputed utility scores were then used to calculate imputed 12-month QALYs using linear interpolation (40).

While the descriptive analysis suggested the data can be described as MAR as missingness can be accounted for using the observed data (employment and level of educational attainment), this is never certain given we cannot observe which unobserved factors we may be missing. As such, to evaluate the uncertainty around this assumption and avoid bias, it is best practice to explore how the results may change if we assume the data are MNAR. Leurent et al. (40) recommend conducting scenario analysis around the imputed values, and as such we apply a utility decrement of varying severity based on whether the participant has been readmitted to acute care. The multiple imputation process was repeated but with a utility decrement weighting applied to the imputed utilities so that the imputed utility was multiplied by 0.9 if the participant had been readmitted to acute care in scenario 2, 0.8 in scenario 3 and 0.7 in scenario 4. Scenario 1 is the MAR scenario where no utility decrement is applied.

#### 2.5.4. Sensitivity analysis around resource use skew

The costs associated with healthcare resource use are often skewed, with a high number of participants accumulating at very low or zero values, and is certainly the case here due to the high costs associated with readmission. Therefore using TSB, we estimate a generalized linear model (GLM) using a gamma distribution to evaluate how accounting for this pattern in resource use costs may impact the cost-effectiveness results using the MICE data set.

All analyses were conducted in Stata 16.

## 3. Results

### 3.1. Baseline characteristics

Baseline characteristics are shown in Table 1. Participants are split into those with complete utility data and those missing utility data at one or more time points, to begin investigating whether there are any significant differences between these groups and if this varies between the intervention and control group. There is no evidence to suggest that there are any significant differences between the four groups at baseline.

**Table 1:** Comparison of sample characteristics at baseline.

Characteristic	Complete utility data (*N* = 223)		Missing utility at one or more timepoints (*N* = 218)	
	Intervention (*N* = 107)	Control (*N* = 116)	Intervention (*N* = 114)	Control (*N* = 104)
Male sex: *n* (%)	43 (40)	46 (40)	47 (40)	42 (40)
Age: mean years (SD)	46 (13)	46 (12)	46 (14)	46 (13)
Ethnicity: *n* (%)				
White (UK and non-UK)	63 (59)	77 (66)	80 (70)	62 (60)
Black (UK, African, Caribbean, mixed, and other)	24 (22)	23 (20)	21 (18)	20 (19)
Asian (UK, South Asian, Chinese, mixed, and Other)	8 (7)	7 (6)	7 (6)	6 (6)
Other	12 (11)	7 (6)	6 (5)	13 (13)
UK born	79 (74)	89 (76)	97 (85)	75 (72)
Marital status: *n* (%)				
Single	62 (58)	74 (64)	79 (70)	71 (68)
Married or cohabiting	27 (25)	31 (27)	19 (16)	21 (20)
Separated or divorced	16 (15)	11 (9)	11 (12)	12 (12)
Widowed	2 (2)	0 (0)	5 (4)	0 (0)
Lifetime admissions to psychiatric hospital: *n* (%)				
Never	67 (63)	72 (62)	67 (59)	60 (58)
1	15 (14)	20 (17)	12 (11)	18 (18)
2–5	18 (17)	19 (16)	21 (18)	21 (20)
> 5	7 (7)	5 (4)	14 (12)	5 (5)
Periods of support from crisis resolution teams				
1	58 (54)	54 (47)	53 (46)	48 (46)
2	20 (19)	23 (20)	23 (20)	20 (19)
3–5	20 (19)	24 (21)	27 (24)	26 (25)
6–10	6 (6)	7 (6)	6 (5)	5 (5)
>10	3 (3)	8 (7)	5 (4)	6 (6)

SD: Standard deviation.

### 3.2. Costs and effects

#### 3.2.1. Cost of the intervention

PSWs are costed at £25 per hour (32). PSW supervision varied in frequency and grade of clinical staff providing the supervision. Supplementary Table 1 shows a comparison of the costs depending on whether supervision was weekly or fortnightly. The most common structure was a fortnightly session with a grade 8 supervisor. Therefore, to calculate the cost per PSW, sessions were assumed to be fortnightly, and the cost was weighted for supervisor seniority. Including overheads, the cost of training and supervision per PSW was £2,548. On average, each PSW was allocated 6.5 participants, which equated to a cost per participant in the intervention group of £392.

Participants on average had 5.8 (95% CI 5.3–6.3) appointments with their PSW. According to the intervention manual, each appointment was scheduled to last an hour, at a cost of £25 per hour of PSW time, the average cost of appointments per patient was £145 (95% CI £131 to £159). The total mean cost per participant of the intervention including training and supervision was £537 (95% CI £523 to £551). The cost of the workbook was not included given both groups received it.

#### 3.2.2. Cost of 12-month mental health service use

Table 2 reports the mean cost of mental health service use at baseline and 12 months for both the intervention group and the control group. The total cost of mental health services at 12 months, adjusting for baseline differences was £6,586 (95% CI: £4,922–£8,249) for the intervention group and £6,605 (95% CI: £4,951–£8,259) for the control group. Including the cost of the intervention and adjusting for baseline, the complete-case mean incremental cost of the intervention group compared with the control group at 12 months was -£261 (95% CI: £2,450–£1928).

**Table 2:** Mean costs and 95% CIs for mental healthcare resource use.

		Intervention		Control	
		Baseline	12 months	Baseline	12 months
Acute care costs	Mean	£6,008	£3,673	£5,351	£4,023
	95% CI	£4,631 to £7,385	£2,156 to £5,220	£3,846 to £6,855	£2,525 to £5,522
Community costs	Mean	£1,740	£2,390	£1,941	£2,581
	95% CI	£1,362 to £2,119	£1,954 to £2,825	£1,478 to £2,405	£2,076 to £3,086
Total	Mean	£7,748	£6,586	£7,292	£6,605
	95% CI	£6,328 to £9,260	£4,923 to £8,949	£5,614 to £8,970	£4,951 to £8,259

#### 3.2.3. QALYs

Mean unadjusted utility scores generated from participant-completed EQ-5D-3L are reported in Table 3. The four participants who died during the trial are included; these were all in the control group. The mean QALYs at 12 months, for which the utility value was taken by drawing a straight line between 4 and 18 months (shown in Supplementary Figure 1), were 0.651 (95% CI 0.612 to 0.689) for the intervention group and 0.640 (95% CI 0.600 to 0.679) for the control group, a mean difference of 0.011 (95% CI: −0.043 to 0.065). The mean QALYs at 18 months, adjusted for baseline and discounted at 3.5% per year after 12 months, were 0.991 (95% CI: 0.931–1.051) for the intervention group and 0.968 (95% CI: 0.907–1.03) for the control group. The mean difference between the two groups was 0.023 (95% CI: −0.062 to 0.107).

**Table 3:** Mean utility scores generated from the EQ-5D-3L and unadjusted 12- and 18-month QALYs. 3.5% discounting for utility scores over 12 months.

		Intervention	Control
Baseline	*N*	217	220
	Mean (SD)	0.613 (0.323)	0.595 (0.331)
	*N* missing (%)	4 (2)	0 (0)
4 months	*N*	173	169
	Mean (SD)	0.670 (0.310)	0.658 (0.328)
	*N* missing (%)	48 (22)	51 (23)
18 months	*N*	122	124
	Mean (SD)	0.698 (0.331)	0.675 (0.322)
	*N* missing (%)	99 (45)	96 (44)
12 months QALYs	*N*	107	116
	Mean (SD)	0.664 (0.271)	0.627 (0.308)
	*N* missing (%)	114 (52)	104 (47)
18 months QALYs	*N*	107	116
	Mean (SD)	1.011 (0.403)	0.950 (0.450)
	*N* missing (%)	114 (52)	104 (47)

### 3.3. Cost-effectiveness—original primary analysis

**Figure 1:**
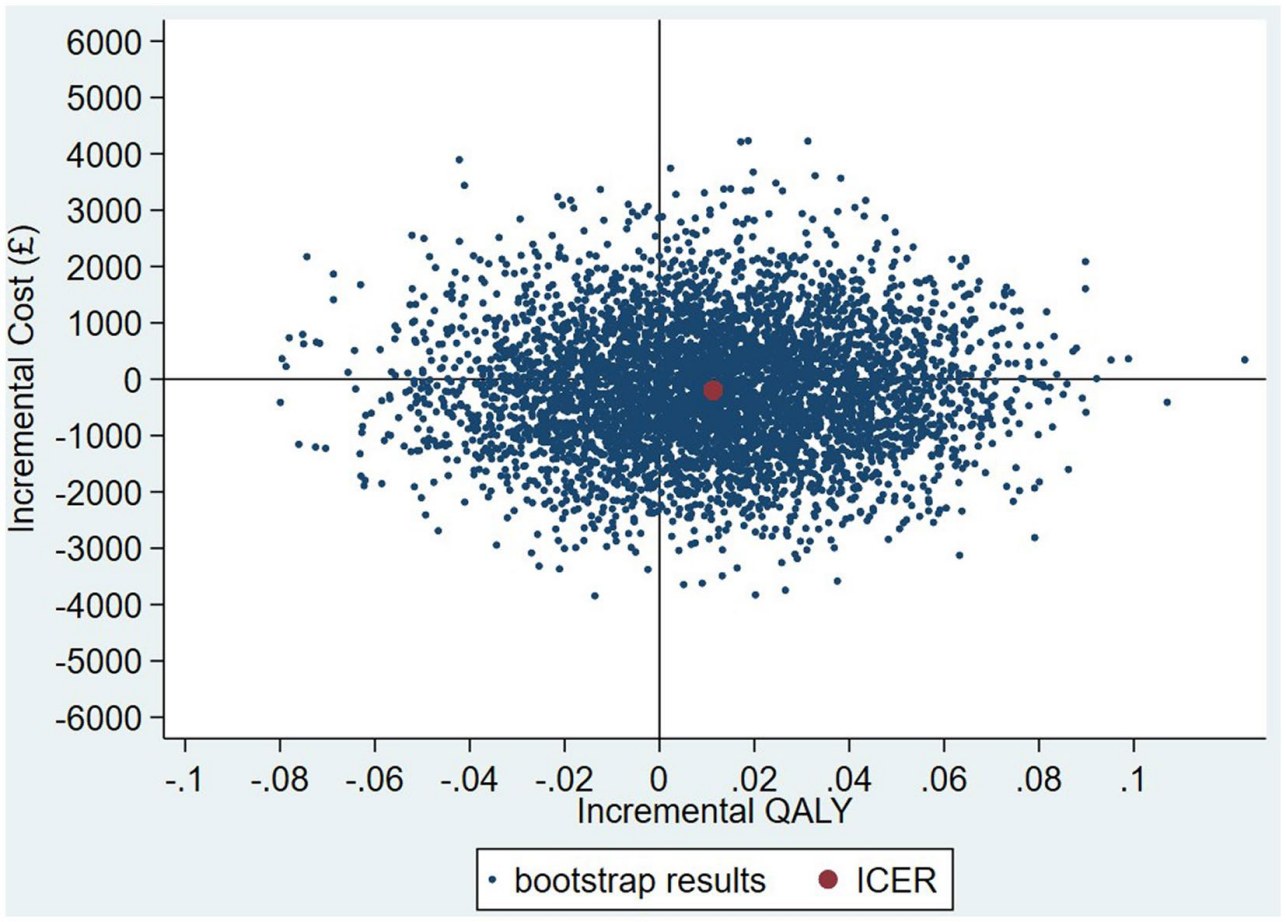
Cost-effectiveness plane (CEP) for 12-month QALYs and 12-month costs based on running separate bootstrap regressions for costs and QALYs (*N*_costs_ = 441/441, *N*_QALYs_ = 223/441).

**Figure 2:**
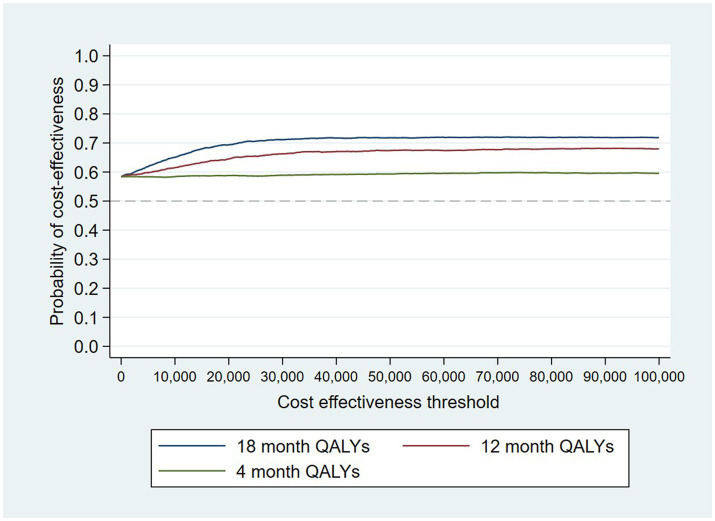
Cost-effectiveness acceptability curves (CEACs) for 4-, 12-, and 18-month QALYs based on running separate bootstrap regressions for costs and QALYs (*N*_costs_ = 441/441, *N*_QALYs_ = 223/441).

The intervention dominates the control group as it results in more QALYs and lower costs, although the differences were not significant. Figure 1 shows the CEP using the 12-month QALYs and 12-month costs from the original analysis. The CEAC in Figure 2 reports the probability of cost-effectiveness at different thresholds using 12-month costs with 4 and 18-month QALYs and 12 months calculated as a linear change between 4-and 18-month QALYs.

At a cost-effectiveness threshold of £20,000 per QALY gained, the probability that the intervention was cost-effective compared to the control was 65% based on 12-month QALYs calculated using linear interpolation. The probability of the intervention being cost-effective compared to control increases as the duration of follow-up increases (see Figure 2). This occurs from a combination of the maximum QALYs achievable increasing with a longer follow-up duration and the difference in utility between the two groups appearing to persist through time. This is in addition to the costs remaining constant as we do not have any costs past 12 months.

### 3.4. Nonparametric two-stage bootstrap

**Figure 3:**
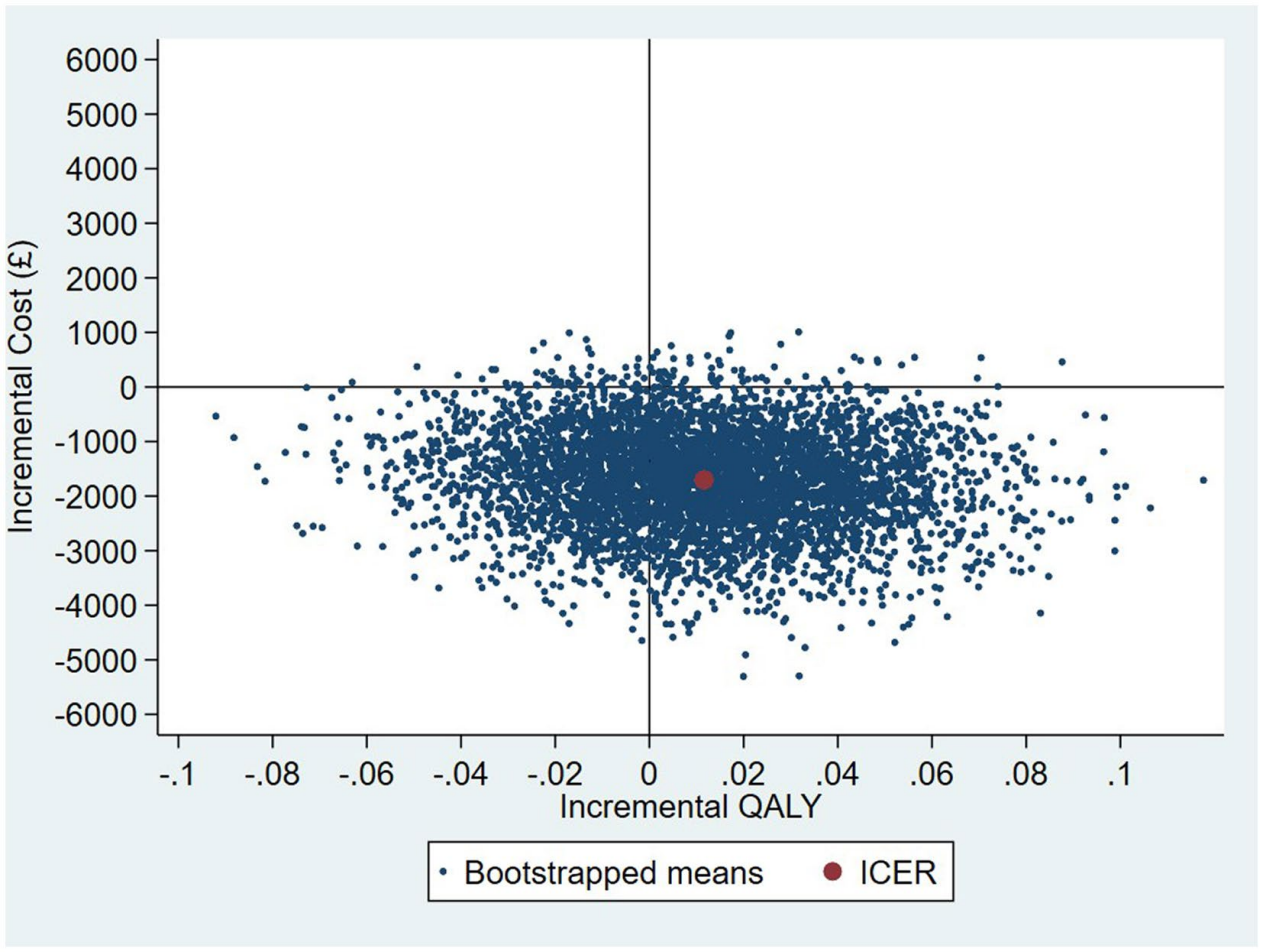
CEP for 12-month QALYs (using TSB method, *N* = 223/441).

**Figure 4:**
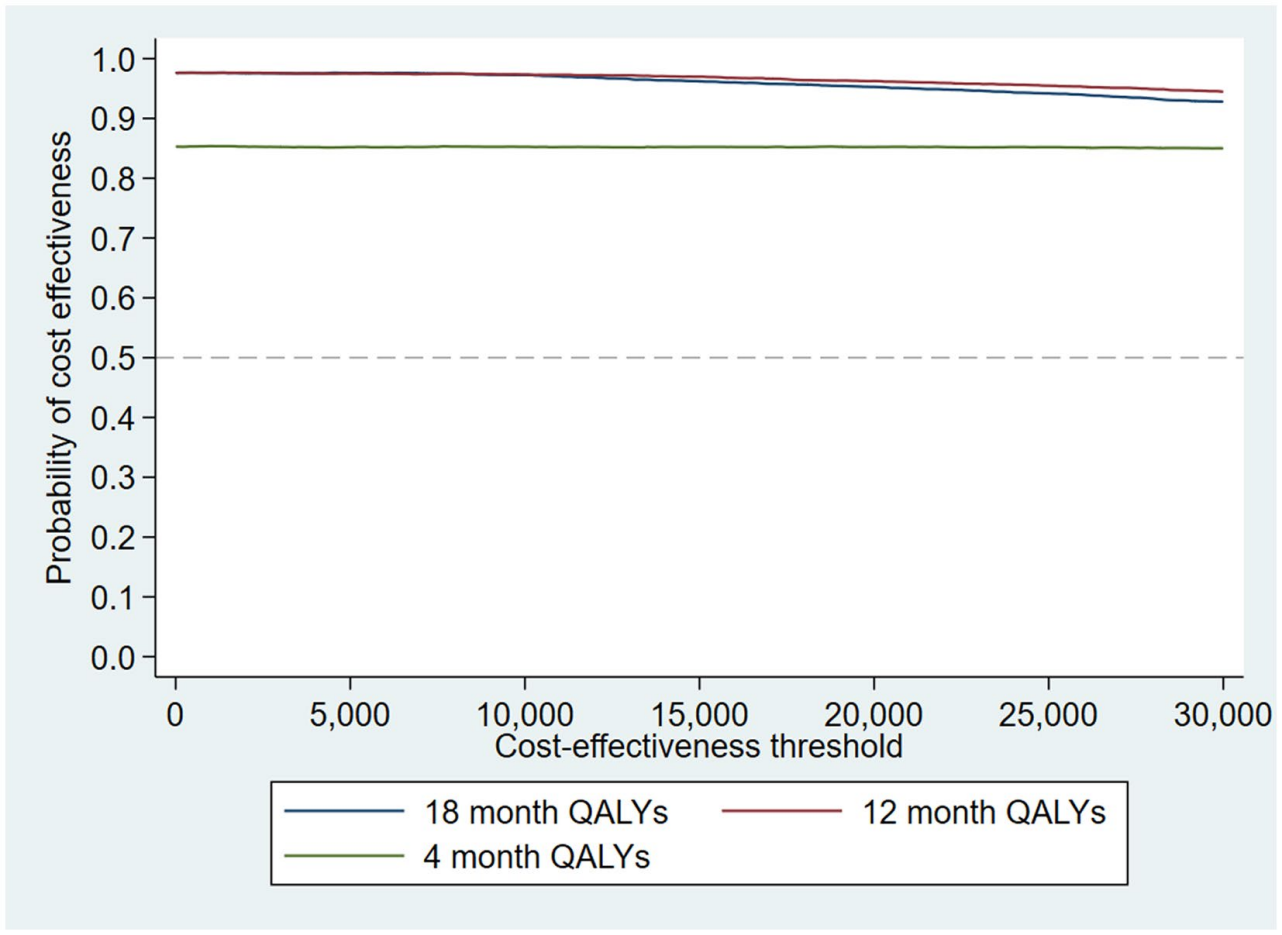
CEAC using TSB method (*N* = 223/441).

The results of the TSB are shown in Figures 3, 4, showing the results on a CEP and CEAC, respectively. Using 12-month QALYs calculated using linearly interpolated utility at 12 months, the intervention is 96% cost-effective at a threshold value of £20,000 per QALY. Comparing the results from the CEP in Figure 3 to those in Figure 1, the CEP for the separate regressions, illustrates that this is because, for the TSB, the majority of bootstrap iterations lie in the bottom two quadrants (cost-saving). Despite the apparent advantage provided by the TSB of accounting for the relationship between costs and outcomes by sampling costs and QALYs at the same time, the analysis is potentially biased as it only includes costs for trial participants who have complete utility data (*N* = 223/441), hence missing many individuals. Table 4 shows how costs differ between those with complete and incomplete utility data across the two groups. Those with missing utility data have significantly higher acute care costs at 12 months than those with complete utility data [£5,855 (95% CI: £3,888–£7,822) vs. £1885 (95% CI: £1,045–£2,725); *p* < 0.001].

**Table 4:** Mean cost of mental healthcare resource use for those with complete and incomplete utility data.

		Complete utility data (*N* = 223)		Missing utility data at one or more time points (*N* = 218)	
		Intervention (*N* = 107)	Control (*N* = 116)	Intervention (*N* = 114)	Control (*N* = 104)
Acute care costs baseline	Mean (SD)	£5,639	£3,980	£6,356	£6,879
	95% CI	£3,588 to £7,689	£2,328 to £5,631	£4,476 to £8,235	£4,284 to £9,474
Community costs baseline	Mean	£1,314	£1,507	£2,141	£2,427
	95% CI	£928 to £1,699	£1,079 to £1,936	£1,505 to £2,777	£1,571 to £3,282
Total acute care costs 12 months	Mean	£1,122	£2,589	£6,067	£5,624
	95% CI	(£440 to £1,805)	(£1,101 to £4,077)	(£3,184 to £8,949)	(£2,926 to £8,322)
Total community care costs at 12 months	Mean	£1,888	£2,212	£2,861	£2,993
	95% CI	(£1,393 to £2,382)	(£1,628 to £2,796)	(£2,159 to £3,563)	(£2,144 to £3,843)

### 3.5. Uncertainty in 12-month estimated QALYs (using TSB)

**Figure 5:**
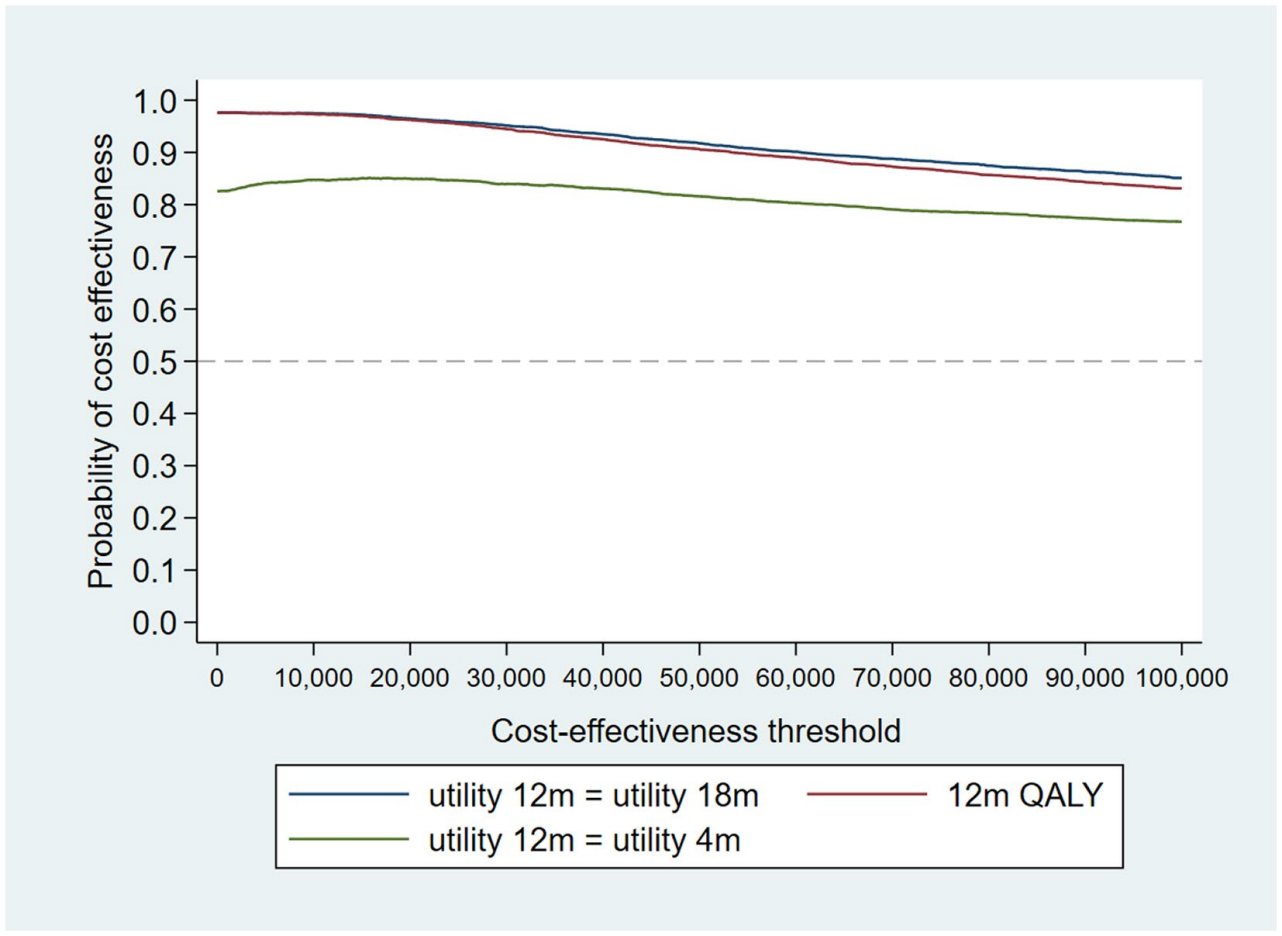
CEAC for sensitivity analysis of 12-month QALYs (using TSB method, *N* = 223/441).

When the analysis was replicated using utility at 4 months to calculate 12-month QALYs using last value carried forward, the probability that the intervention was cost-effective compared to TAU fell to 85% at a cost-effectiveness threshold of £20,000/QALY gained. The analysis using utility at 18 months to calculate 12-month QALYs using next value carried backward had very similar results to the analysis using linearly interpolated utility at 12 months. This suggests that the results are driven by an improvement in recorded utility at 18 months (Table 3) rather than simply having more QALYs available and hence a larger potential incremental benefit. The CEAC is shown in Figure 5.

### 3.6. Missing data analysis

#### 3.6.1. MAR analysis

**Figure 6:**
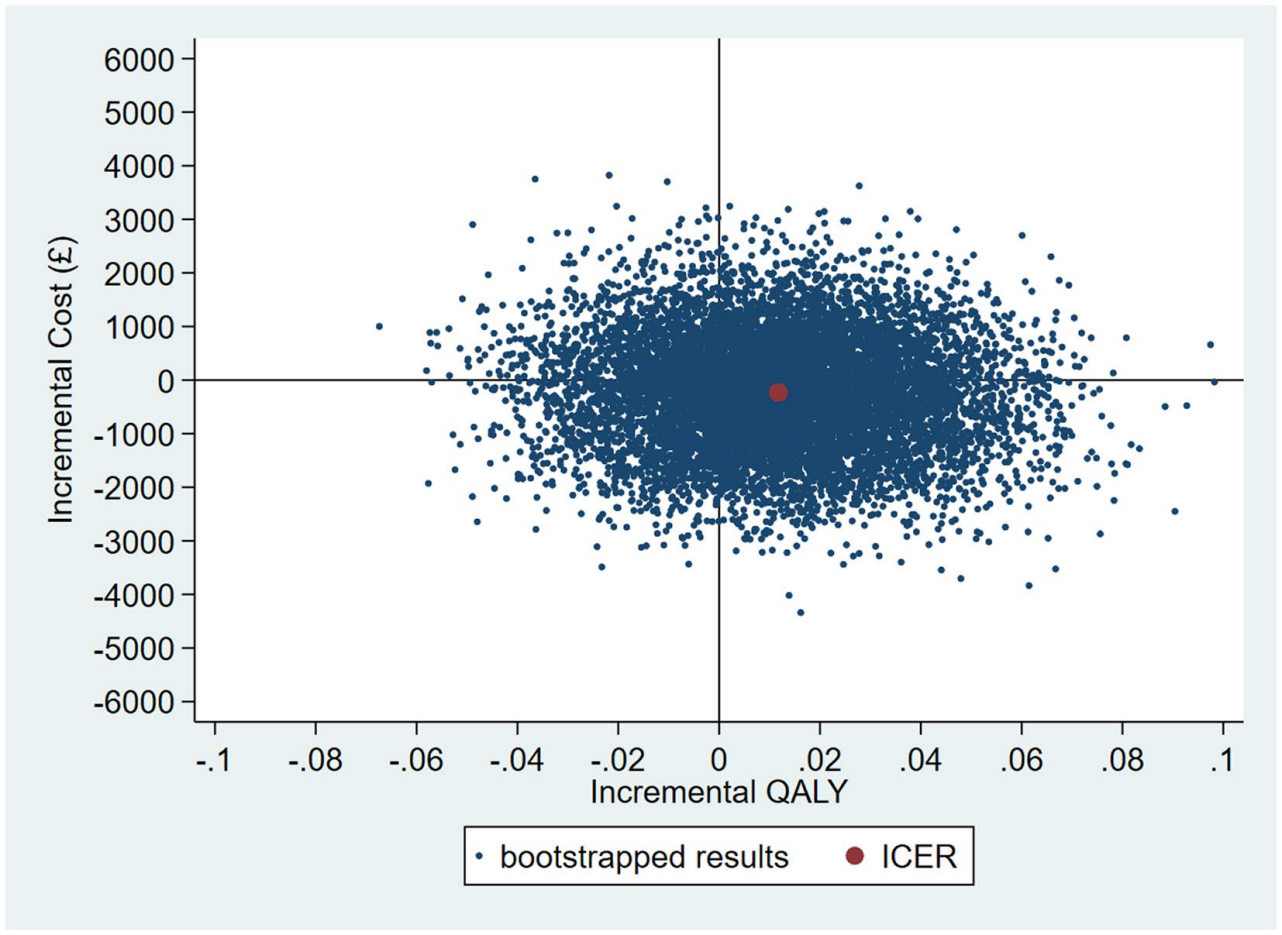
CEP MAR using 12-month QALYs and TSB method (using MICE data).

**Figure 7:**
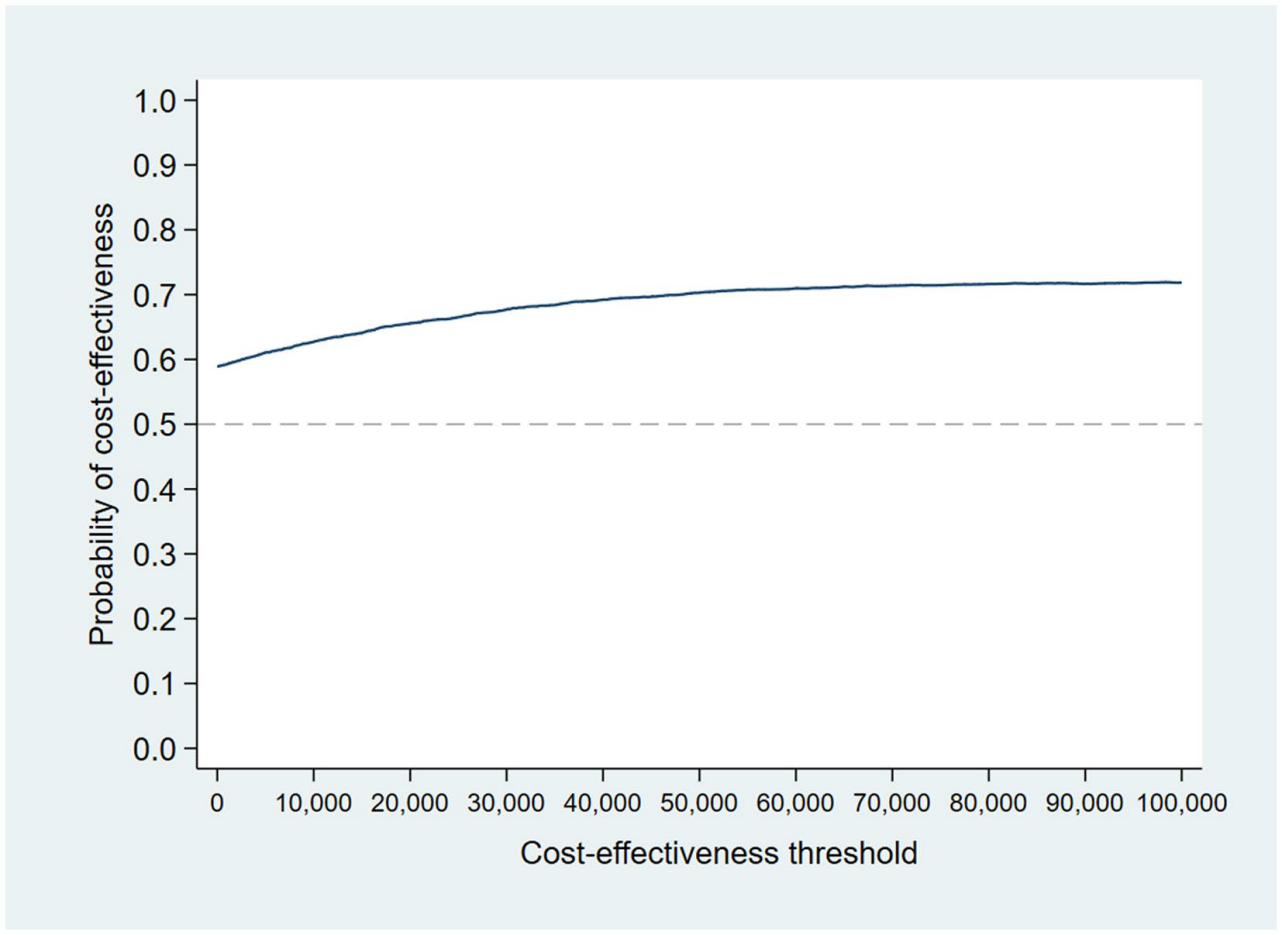
CEAC MAR using 12-month QALYs and TSB method (using MICE data).

Following multiple imputation, the mean difference in QALYs between the intervention and control group at 12 months was 0.012 (95% CI: −0.033 to 0.057). The CEP and CEAC were constructed using the TSB following multiple imputation, and are shown in Figures 6, 7. The probability that the intervention was cost-effective compared to the control was 66% at a cost-effectiveness threshold of £20,000/QALY gained.

#### 3.6.2. MNAR sensitivity analysis

The example provided by Leurent et al. (40) experiments with applying different weights to the imputed utility in different scenarios based on the assumption that those with missing utility data may be systematically worse off. This is likely to be the case here given those missing utility data have significantly higher acute care costs indicating they are in worse health than those with complete utility data. However, unlike the example in which they apply different weights to the treatment and control group, we apply a different weight based on whether the participant has been readmitted to acute care. Logistic regression showed that those who were readmitted to acute care within 1 year were 20% more likely to have missing utility data at 4 months and 7% more likely to be missing utility data at 18 months compared with those who were not readmitted. It is plausible that those who were missing utility data and had been readmitted to acute care had a lower health-related quality of life.

The probability of the intervention being cost-effective compared with TAU increased as the utility decrement weighting increased. All four scenarios are presented on a CEP in Supplementary Figure 2. The results were very close to those found in the MAR analysis with the probability of cost-effectiveness ranging from 64.7% (MAR) to 66.4% (imputed utility multiplied by 0.7 if the participant has been readmitted) at a threshold value of £20,000/QALY gained, shown on a CEAC in Supplementary Figure 3.

#### 3.6.3. Accounting for resource use skew

**Figure 8:**
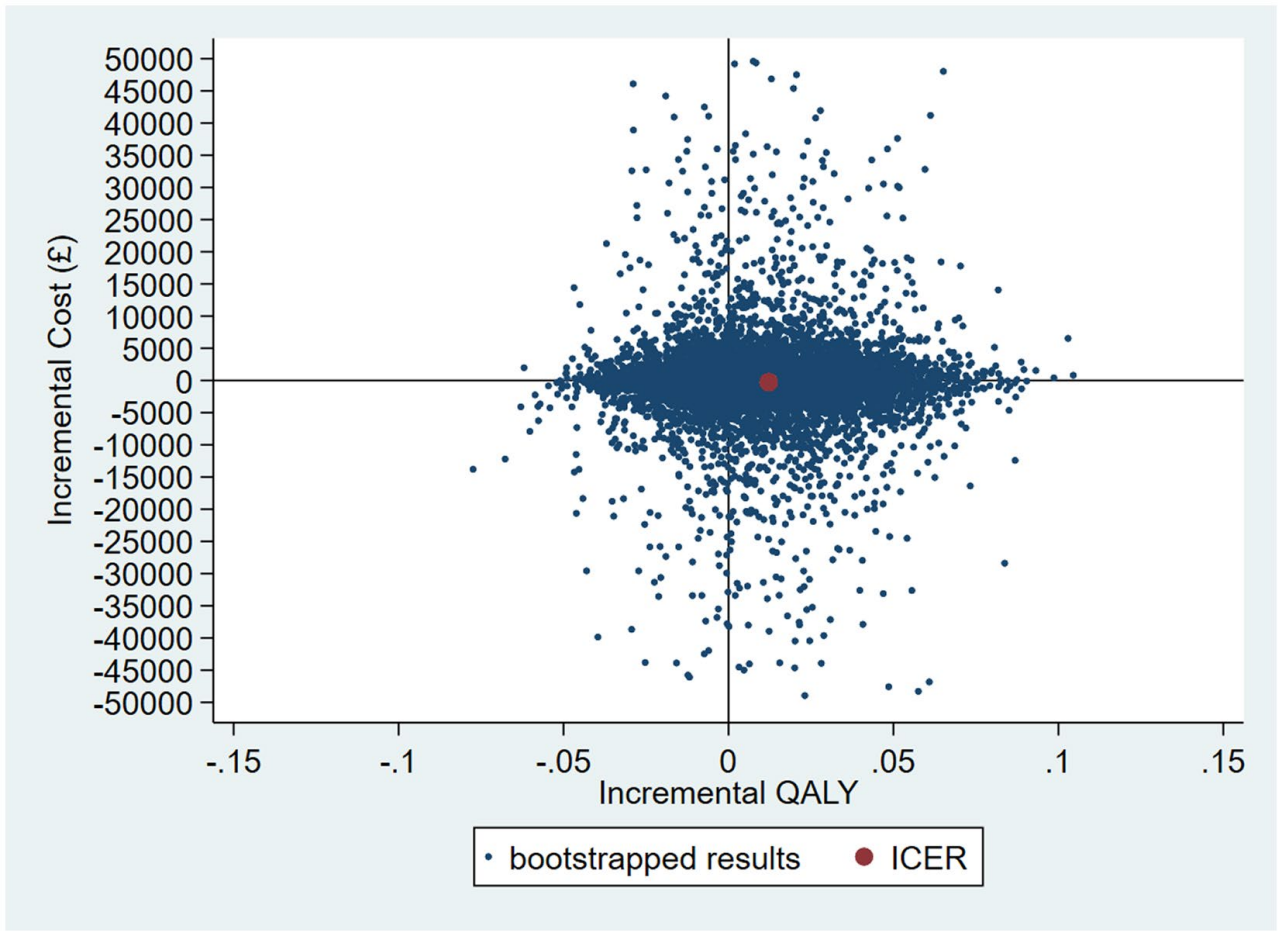
CEP for GLM model accounting for resource use skew using 12-month QALYs and TSB method applying a gamma distribution (using MICE data).

**Figure 9:**
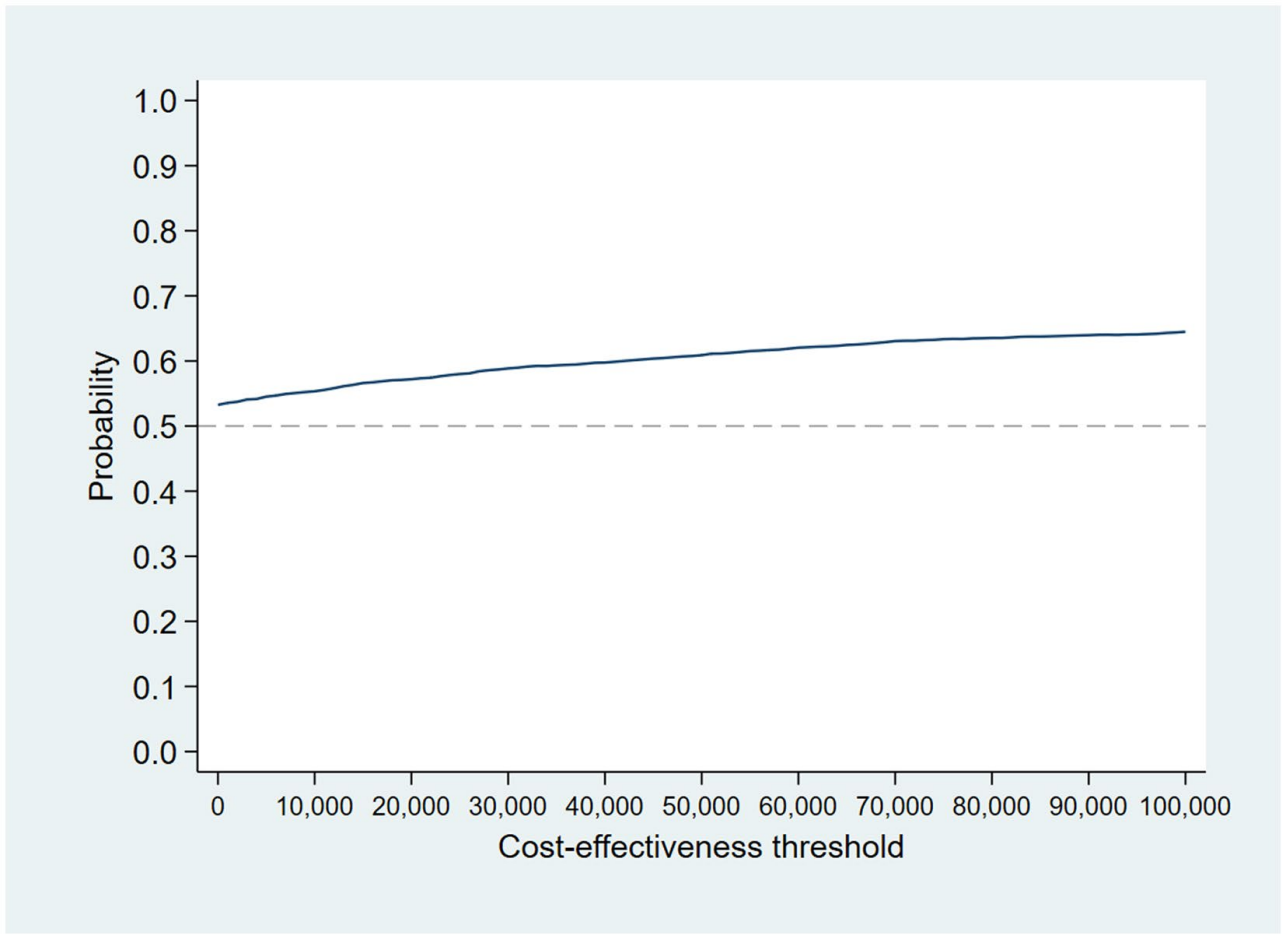
CEAC for GLM model accounting for resource use skew using 12-month QALYs and TSB method applying a gamma distribution (using MICE data).

Accounting for the skew in the resource use cost data by using a GLM model, the probability that the intervention was cost-effective compared with control is 57% at a cost-effectiveness threshold of £20,000 per QALY gained (see Figures 8 and 9 for the bootstrapped results illustrated on a CEP and CEAC). The mean cost difference is –£427, with 90% of iterations from the bootstrap falling between -£9,186 and £8,522.

Table 5 summarizes the probability of cost-effectiveness for each analysis for ease of comparison.

**Table 5:** Summary of results from each analysis.

Analysis	Probability at £20,000	Probability at £30,000
Original analysis	65%	66%
Non-parametric TSB	96%	94%
MI MAR	65%	69%
MI MNAR Utility = imputed utility × 0.8 for those missing & readmitted	66%	69%
GLM MAR	57%	59%

## 4. Discussion

The aim of this study was to determine whether a peer-supported self-management intervention delivered by PSWs was cost-effective compared with a self-management workbook plus TAU control. As we had complete data for resource use at 12 months and baseline, and self-report data for utilities at baseline, 4 and 18 months with a large proportion of missing data, we conducted a range of analyses to evaluate the impact of conducting more complex analyses on the results. The intervention dominated the control, as it cost less and yielded more QALYs, although this difference was not significant and had wide confidence intervals. Both the complete-case linear regression and MAR multiple imputation analysis had a probability of 65% that the intervention was cost-effective compared to control at a £20,000/QALY cost-effectiveness threshold over 12 months. This increased to 69% if 18-month utility data and 12-month costs were used as the intervention had a sustained health-related quality-of-life increase.

Resource use came from mental health service use only, and as this was collected from patient records the analysis benefitted from a high level of follow-up for resource use (intervention = 218/221, control = 216/220). This meant that the cost perspective of the analysis was limited to mental health costs only. As the probability that the intervention was cost-effective increased with increasing follow-up periods from 4 to 18 months, this suggests that the benefit of the intervention may be maintained over time, potentially increasing the probability that the intervention is cost-effective through increased QALYs and cost-savings. Given the different follow-up duration for costs and QALYs these results should be interpreted with caution.

This analysis brings into perspective the importance of parsimony when choosing evaluation methods. Given that healthcare costs and health-related quality of life are intrinsically linked, it is sensible that we should seek to use methods which take this relationship into account when assessing the cost-effectiveness of a treatment. This, however, requires that both resource use and preference-based health-related quality of life information are present to calculate costs and QALYs, respectively. The results of the complete-case TSB provide evidence of the possible bias that can be introduced when, in this case, information for the denominator of the ICER (ICER = difference in costs/difference in outcome) is missing, restricting the number of cases available for the numerator. Here, analyses using complete case and multiple imputation of utility values are consistent in suggesting the intervention is cost-effective compared to control at 12 months, with a 65% probability that the intervention is cost-effective at a cost-effectiveness threshold of £20,000 per QALY gained. This decreased to 57% when the distribution of the data was taken to account. It is clear in this example that the complete-case TSB leads to an over-estimate of cost-effectiveness and if used incorrectly in other similar analyses, it could lead to an intervention which is not cost-effective being recommended for use, or to not recommending an intervention due to underestimating the cost-effectiveness resulting in patients not receiving the best care available. The results of this analysis show that, when the level of missing data is heavily unbalanced between costs and outcomes, multiple imputation can allow us to implement the preferred method while avoiding introducing bias into the results.

### 4.1. Strengths and limitations

This analysis was based on data from a randomized control trial in mental healthcare Trusts in England, and provides a robust estimate of the cost-effectiveness of the intervention in this setting. We had relatively complete follow-up for mental health service use data, although the choice of statistical methods for the cost-effectiveness analysis could potentially introduce bias into the analysis when incorporating QALYs, something we have explored in this paper. The cost perspective was limited to specialist mental health services given that this was all that we could obtain from patient files and asking patients to complete questionnaires regarding resource use was considered an onerous addition. Consequently, we are unable to say anything about impact on wider healthcare service resource use or employment and productivity as a result of the trial.

A complete analysis at 18 months was not possible, as although we had EQ-5D-3L data for participants for the calculation of QALYs, we had no resource use information beyond 12 months. A 4 month cost analysis was also not possible because of the way data was collected from clinical records, giving the number of attendances over 12-months, not when they occurred. Given improvements in utility continue through to 18 months, there may be further QALY gains and cost-savings to be made beyond 18 months, potentially further extending cost-effectiveness if these improvements are related to lower admissions and therefore lower costs. As a result the 18-month cost-effectiveness analysis is potentially a conservative one, if one that should be interpreted with caution given the different time horizon for costs and QALYs.

The EQ-5D is potentially not the best outcome measure to have used as it is not as sensitive in serious mental illness (41). Since the trial, a tariff for calculating utility scores from the Recovering Quality of Life (ReQoL) questionnaire has been developed (42). The measure was designed to assess the quality of life of people with different mental health conditions and may be more suitable in future studies of this patient population.

### 4.2. Conclusion

There is a high probability that PSW plus workbook is cost-effective compared to usual care plus workbook for a range of cost-effectiveness thresholds. This is likely to be driven by reduced readmissions (23). The probability of cost-effectiveness though is highly dependent on the statistical methods used for the analysis. As a result, it is important that analysts take into account the potential bias from missing data as part of trials in serious mental illness. We would recommend ensuring that resource use is collected as best as possible from patient files. This needs to be complemented though with methods to ensure minimum loss to follow-up for preference-based measures of health-related quality of life for calculating QALYs to reduce the potential bias in the analysis.

## Data availability statement

The raw data supporting the conclusions of this article will be made available by the authors, without undue reservation.

## Ethics statement

The studies involving human participants were reviewed and approved by London Camden and Islington Research Ethics Committee. The patients/participants provided their written informed consent to participate in this study.

## Author contributions

ML and RH designed, conducted, and interpreted the analyses. SJ and BL led the study. LM and GA provided advice on the analyses and designed, conducted, and interpreted the statistical analyses for the clinical paper. DO and DL helped to design the study. All authors contributed to the paper and approved the final version, and all took responsibility for its content.

## Funding

The paper reports work undertaken as part of the CORE study which was funded by the National Institute for Health Research under its Programme Grants for Applied Research program (reference RP-PG-0109-10078). The views expressed are those of the authors and not necessarily those of the NHS, the National Institute for Health Research, or the Department of Health.

## Conflict of interest

The authors declare that the research was conducted in the absence of any commercial or financial relationships that could be construed as a potential conflict of interest.

## Publisher’s note

All claims expressed in this article are solely those of the authors and do not necessarily represent those of their affiliated organizations, or those of the publisher, the editors and the reviewers. Any product that may be evaluated in this article, or claim that may be made by its manufacturer, is not guaranteed or endorsed by the publisher.

## Supplementary material

The Supplementary material for this article can be found online at: https://www.frontiersin.org/articles/10.3389/fpsyt.2023.1031159/full#supplementary-material
