# Supplementary material for: Cost-effectiveness of peer-supported self-management for people discharged from a mental health crisis team: methodological challenges and recommendations
Source: Front Psychiatry. 2023 Jun 2;14:1031159. doi: 10.3389/fpsyt.2023.1031159 (PMC10272352; doi:10.3389/fpsyt.2023.1031159)
Supplement: Supplementary file 1 [file Data_Sheet_1.docx]

Supplementary Material

# Sensitivity analysis (cost of intervention)

## Mental health trust costs and fortnightly supervision (using TSB method (N=223/441))

The costs per hour of PSW and clinical supervision staff provided by the mental health trusts were lower than those from the PSSRU. Costs were provided as mean values with no accompanying distribution. The Trusts’ costs per hour were £13 and £31 for PSW and clinical supervision respectively, assuming clinical supervision was provided by a Band 8a clinician. The Trusts’ cost per hour of a CRT manager was £25 and £20 per hour for a Band 6 nurse. Using these costs provided by the mental health trusts, the total cost of supervision and training was £1,343 per PSW and £207 per participant in the treatment group. When these costs were applied instead of the PSSRU unit costs, using the TSB, participants in the PSW group cost £437 less than the workbook only group on average (95% CI -£2,205 to £1,331). At a cost-effectiveness threshold of £20,000 per QALY there is a 98% chance that the PSW intervention is cost-effective compared to workbook only with 12-month QALYs and 12-month costs using costs provided by mental health trusts.

## Weekly supervision of PSWs

When supervision was assumed to be weekly instead of fortnightly, the total cost of supervision and training using PSSRU costs was £3,356 per PSW (Supplementary Table 1) and £456 per participant in the intervention group. Using the TSB with these costs, participants in the PSW group cost on average £184 less than those in the workbook only group (95% CI -£1951.19 to +£1,583). Using these costs, the probability that the PSW intervention is cost-effective compared to the control is 95% at a threshold value of £20,000 per QALY using 18-month QALYs and 12-month costs.

# Supplementary Figures and Tables

## Supplementary Figures


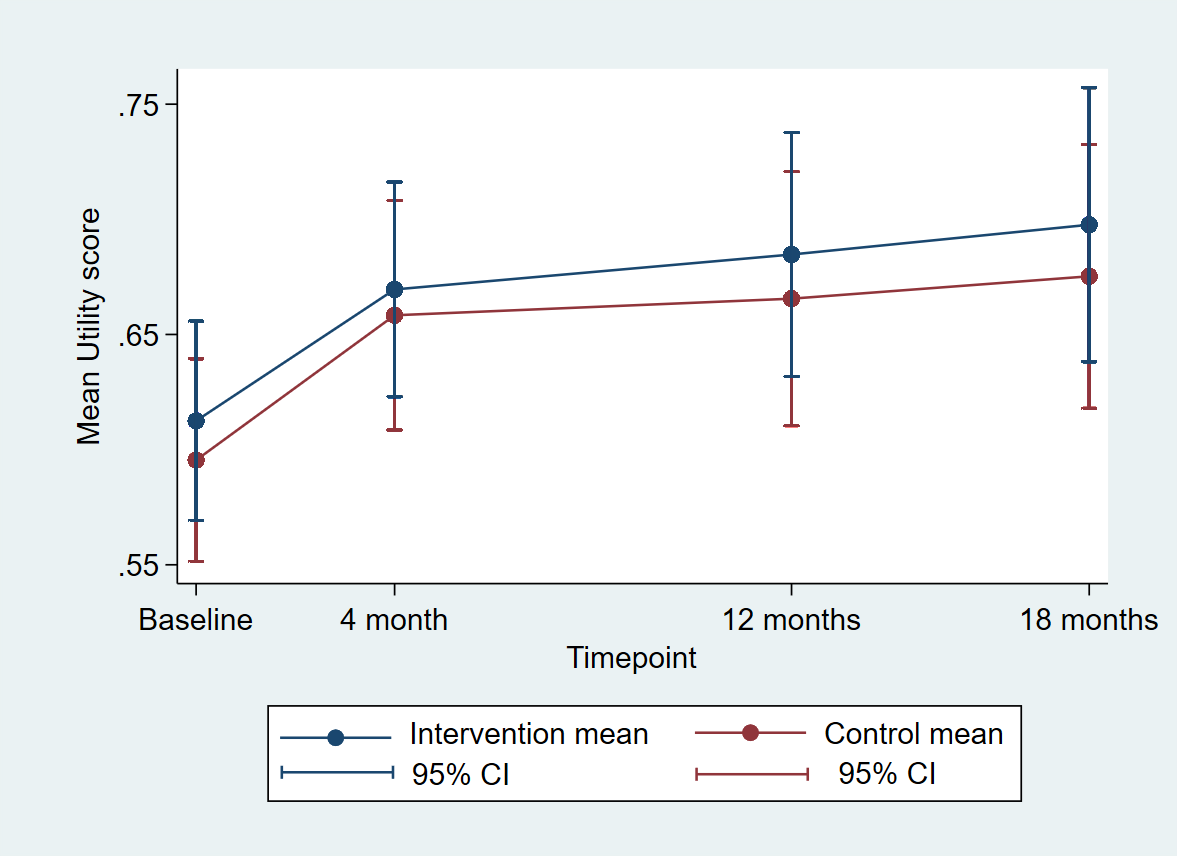
**Supplementary Figure 1. Mean utility by group and accompanying 95% CIs**


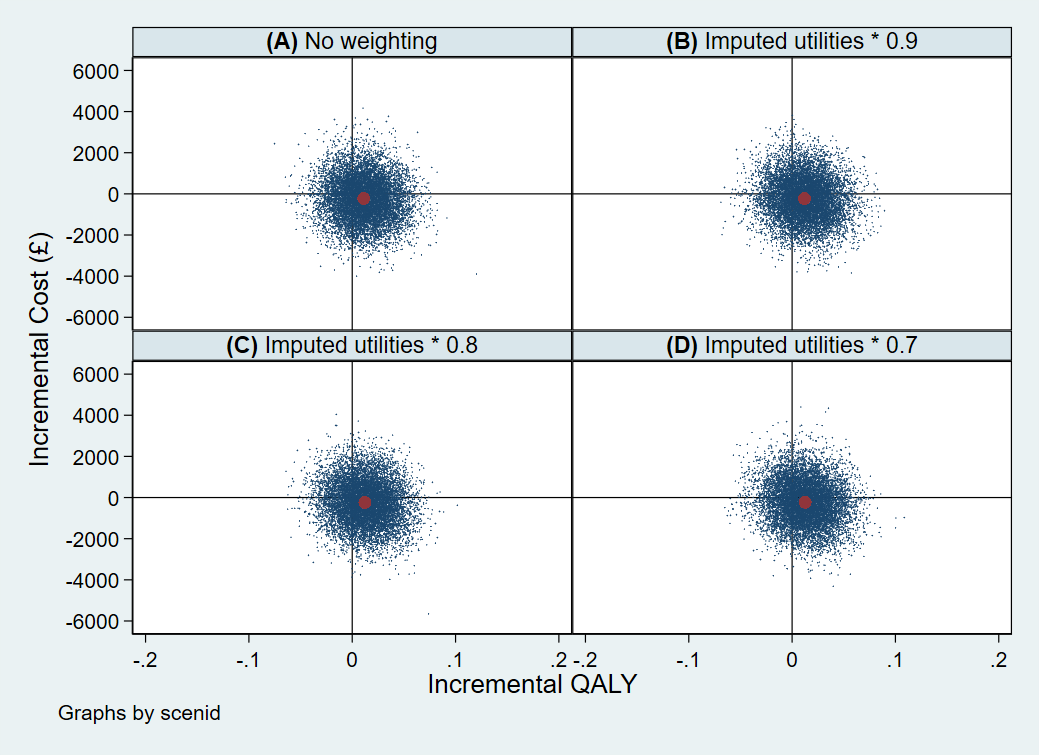


Supplementary Figure 2: CEPs for MNAR scenarios using 12-month QALYs and TSB method (using MICE data)


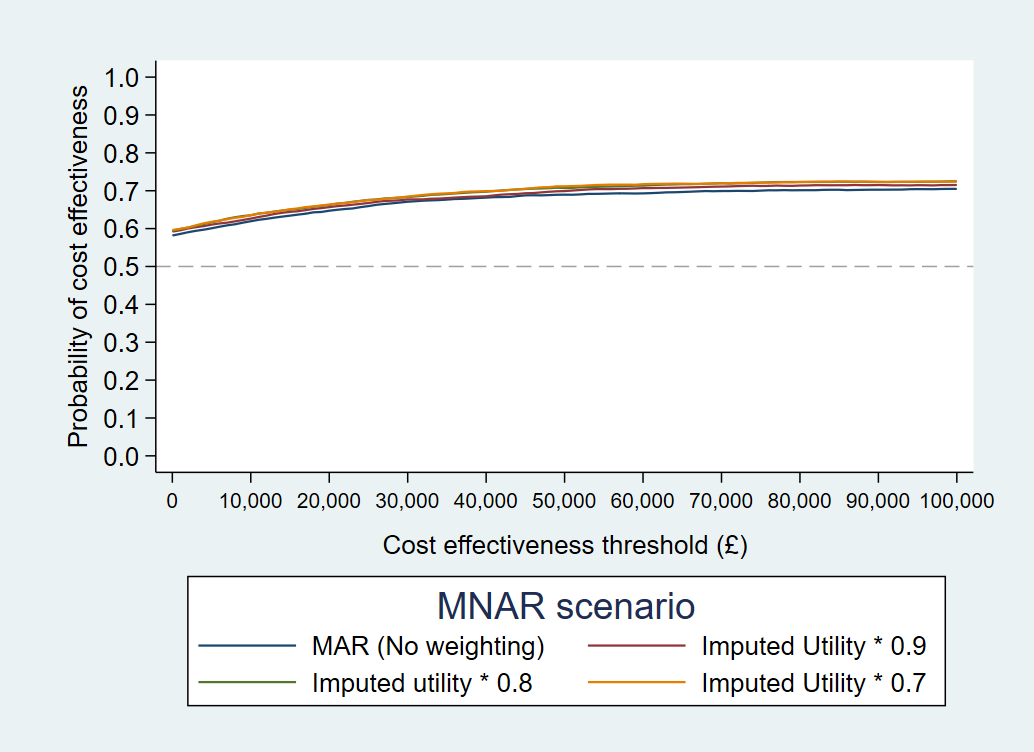


Supplementary Figure 3: CEACs for MNAR scenarios using 12-month QALYs and TSB method (using MICE data)

## Supplementary Tables

Supplementary Table 1: Cost of peer support training and supervision (using 2015/2016 costs)

| Activity | Description | | No. PSW | Total Cost | Cost per PSW | |
| --- | --- | --- | --- | --- | --- | --- |
| *UCL training costs | 7 days’ training plus 4 days’ prep | | 30 | £1,560 | £52 | |
| PSWs attending training | 67.5 hours (9 days) per PSW | | 30 | £50,625 | £1,688 | |
| PSWs Attending fortnightly supervision (*Primary*) | 12 meetings (2 hours per meeting) | | 30 | £18,000 | £600 | |
| Providing fortnightly group supervision (*Primary*) | 12 meetings (2 hours per meeting) | 2 band 8a clinicians (£62/hour) | 15 | £2,976 | £198 | |
|  |  | 1 band 7 CRT manager (£52/hour) | 5 | £1,248 | £250 | |
|  |  | 2 band 6 nurses (£42/hour) | 10 | £2,016 | £201 | |
|  | Weighted cost of supervision per PSW | | |  | £208 | |
| **Total cost of training and supervision per PSW (*Primary*)** | | | |  | **£2,548** | |
| PSWs Attending weekly supervision (*SA)* | 24 meetings (2 hours per meeting) | | 30 | £36,000 | £1,200 | |
| Providing weekly group supervision (*SA)* | 24 meetings (2 hours per meeting) | 2 band 8a clinicians (£62/hour) | 15 | £5,952 | £397 | |
|  |  | 1 band 7 CRT manager (£52/hour) | 5 | £2,496 | £499 | |
|  |  | 2 band 6 nurses (£42/hour) | 10 | £4,032 | £403 | |
|  | Weighted cost of supervision per PSW | | | | £416 | |
| **Total cost of training and supervision per PSW (*SA*)** | | | |  | | **£3,356** |
| *UCL = University College London, SA= Sensitivity Analysis | | | | | | |
